# The Impact of Parental Autonomy Support on Family Adaptation in the Context of “Double Reduction”: The Mediating Role of Parent–Child Communication and Cohesion

**DOI:** 10.3390/bs14070534

**Published:** 2024-06-26

**Authors:** Ruibo Xie, Xuan Wang, Yangguang Ding, Yanling Chen, Wan Ding

**Affiliations:** 1 Parent Education Research Center, The Intelligent Laboratory of Child and Adolescent Mental Health and Crisis Intervention of Zhejiang Province, School of Psychology, Zhejiang Normal University, Jinhua 321004, China; 2 College of Education, Zhejiang Normal University, Jinhua 321004, China

**Keywords:** “Double Reduction” policy, parental autonomy support, parent–child communication, parent–child cohesion, family adaptation

## Abstract

The implementation of the “Double Reduction” policy indicates a significant change in the way households operate, such as through parental education conception and parenting form, in which family adaptation needs particular attention. Parental autonomy support has been evidenced to be related to family adaptation in prior studies. However, the mechanism underlying the relationship between parental autonomy support and family adaptation in the context of “Double Reduction” are not clear enough but remain fascinating. This study aims to explore the process through which parental autonomy support affects the whole family’s adaptation in the context of “Double Reduction” from the perspectives of parent–child behavior and emotions (i.e., parent–child communication and parent–child cohesion). A cross-sectional design based on the questionnaire method was used to collect the characteristics of 4239 adolescent parents (1493 fathers and 3427 mothers; *M*_age_ = 43.20, *SD*_age_ = 22.39) one year after the implementation of the “Double Reduction” policy. In addition, this study also used the retrospective method to obtain the characteristics of parental autonomy support before the “Double Reduction” policy. In the context of “Double Reduction”, the research results found that parental autonomy support can predict family adaptation; parental autonomy support can also influence the whole family’s adaptation through the quality of parent–child interaction. This study reveals the impact mechanism of parental autonomy support on family adaptation under the background of “Double Reduction” in China and provides insights on how to improve the adaptation of the entire family from the perspective of parent–child interaction.

## 1. Introduction

In July 2021, China introduced a “Double Reduction” policy to reduce the excessive burden of homework and off-campus tutoring for compulsory education students. This has brought China’s education sector into a new phase of reduction. With the implementation of the “Double Reduction” policy, the interaction time between parents and children has increased, the role of parents in education has become more prominent, and the forms of family interaction have become more diverse. However, for some families, “Double Reduction” is a sudden stress event, indicating a major change in the way the family operates, and they may not be able to adapt in the short term [1]. According to the Family Adjustment and Adaptation Response Model [2], unconventional stress events may lead to family functional disorders that require timely adaptation to maintain the family’s healthy functioning. In other words, under the sudden macro-policy background of “Double Reduction”, families may fall into turmoil and experience difficulties with parenting methods and parent–child relationships, for example. Therefore, it is necessary to investigate the favorable factors that promote family adaptation after the “Double Reduction”. However, the “Double Reduction” policy has been implemented for two years, but there has been no research on how to help families adapt to the changes under the context of “Double Reduction”.

What needs to be clarified here is that the objective of the “Double Reduction” policy is not only to reduce the students’ homework and off-campus tutoring burden outside of school but, more importantly, to meet the students’ diverse needs and provide students with a diversified growth path [3]. Children need more autonomy support from their parents as they grow, which is especially important for children’s mental health and can help develop youths’ individuality [4,5]. However, the majority of Chinese parents have long been accustomed to the child’s educational content and direction of development, and hence, there is a lack of space for the child to choose. This is also one of the main reasons for vigorously promoting the “Double Reduction” policy.

Previous studies have pointed out that parental autonomy support can promote children’s autonomous development, meet children’s basic psychological needs, and bring positive cognitive, social, and emotional outcomes [6,7]. It is worth noting that some studies have shown that parental autonomy support is not only beneficial to the child but may also affect social systems around the child, especially as the most direct micro-family system affecting children’s growth [8]. This may mean that having parents who automatically support their children may result in families who adapt better to the educational changes brought about by the “Double Reduction” policy. However, there has been little attention given to the impact of parental autonomy support at the family level in previous studies, which mostly focus on children. Therefore, it is necessary to research the effect of parental autonomy support on family adaptation and understand the mechanism of action between them in the context of the “Double Reduction” policy.

In addition, implementing the “Double Reduction” gives family members more time to be together and increases the opportunities for interaction between family members. A survey of “Double Reduction” showed that 82.6% of parents of 10,185 primary and secondary school students believed that under the background of “Double Reduction”, the time spent between parents and children increases, which further affects parent–child relationships and greatly promotes parent–child interaction [9]. Previous studies have pointed out that parent–child behavior and emotional interaction have important and special functions in the family, and parent–child behavior interaction can further promote parent–child emotional interaction [10]. Specifically, parent–child communication behavior is an important manifestation of parent–child behavioral interaction, in which parents and children interact with each other and are associated with positive outcomes such as family intimacy and adaptation [11,12,13]. In terms of emotional interaction, parent–child affinity reflects the degree of supportive interaction within the parent–child system and is a significant predictor of children’s emotional connection with their parents [14]. These two may be important internal mechanisms for parental self-support and family adaptation. However, there have been no studies to investigate the effects of parent–child communication and parent–child cohesion in parental autonomy support on family adaptation in the “Double Reduction” environment. Based on this, this study intends to explore the mechanism of parent–child communication and parent–child cohesion in parental autonomy support and family adaptation under the background of “Double Reduction” from the perspective of parent–child behavior and emotional interaction.

### 1.1. The Impact of Parental Autonomy Support on Family Adaptation in the Context of the “Double Reduction”

Parental autonomy support may have an impact on family adaptation. Olson et al. [15] argued that family adaptation can assess the health and stability of a family by measuring the strength of relationships between members of the family and their ability to cope with challenges. In the context of China’s “Double Reduction” policy, family adaptation refers to the ability of the family to change according to the family situation and the family development phase under the “Double Reduction” policy, which includes adaptation of family education, adaptation of the parent–child relationship, adaptation of parents themselves, and adaptation of the marital relationship in four aspects. From a family perspective, parental autonomy support refers to the parenting style in which parents cultivate children’s intrinsic motivational resources and behavioral self-determination [16]. According to the self-determination theory, satisfying the need for autonomy can better stimulate an individual’s intrinsic motivation. An autonomous environment is beneficial for children to explore themselves, form their own emotions and value systems, and promote their adaptation to changes in the environment [6]. In the context of China’s “Double Reduction” policy, parental support for children’s autonomy can help them better adapt to the changes brought about by the educational environment and thus promote the entire family’s adaptation to the “Double Reduction” policy. There are also studies indicating that parental autonomy support, as a positive parenting approach, can promote family relationships, maintain the functioning of the family system, and better respond to external changes (e.g., changes caused by the “Double Reduction”) [7,17]. Therefore, we believe that parental autonomy support may have a direct impact on family adaptation in the context of “Double Reduction”. However, previous studies have lacked direct exploration of the relationship between parental autonomy support and family adaptation, and we do not yet know whether the impact of parental autonomy support on family adaptation will change before and after the implementation of the “Double Reduction” policy.

### 1.2. The Mediating Role of Parent–Child Communication

However, parental autonomy support may also indirectly affect family adaptation, and exploring the potential mechanisms of parental autonomy support’s impact on family adaptation in the context of “Double Reduction” is of great significance. According to family system theory, the family is made up of both individual and interacting subsystems that interact with one another [18]. The mode and quality of parent–child interaction are the core of the internal operation of the family, and parental parenting behavior mainly affects the adaptation of the entire family through parent–child interaction [19]. Parent–child communication is the process by which parents and adolescents exchange information, perspectives, attitudes, emotions, and other content to solve problems or enhance emotional connections [20]. Research has shown that parental parenting behavior can directly or indirectly affect the mode and quality of parent–child communication [21]. Scientific educational concepts such as giving children autonomy and providing support can promote the formation of good parent–child communication patterns and improve communication quality [22,23]. In addition, studies have found that parent–child communication helps families better adapt to the “Double Reduction” policy [24]. Good communication patterns can promote mutual understanding and support among family members, thus helping the whole family to better withstand and cope with the new changes in the external education reform and ultimately obtaining family adaptation [25]. In summary, in the context of “Double Reduction”, parental autonomy support may affect the family’s adaptation level by influencing parent–child communication. However, no research has yet explored this internal mechanism based on the background of “Double Reduction”.

### 1.3. The Mediating Role of Parent–Child Cohesion

Parent–child cohesion, a vital indicator of the quality of parent–child relationships, is commonly described as the intimate emotional bonding between children and their parents [26]. The implementation of the “Double Reduction” policy has brought both opportunities and pressure to the entire family. In this context, parent–child cohesion plays an important role in providing suitable conditions for the physical and mental development of members [27]. Research has shown that parent–child cohesion is beneficial for parents and children to communicate together and cope with external difficulties or challenges, helping families adapt to changes brought about by the external environment [28]. When facing the pressure and challenges under the “Double Reduction” policy, family members with higher levels of parent–child cohesion may have stronger adaptability. At the same time, parent–child cohesion is also influenced by the parenting style of parents [29,30]. Research has shown that self-supporting parenting can strengthen parent–child relationships and cultivate children’s attachment to their parents [8]. Parents adopting a self-supporting parenting approach can improve parent–child cohesion, thereby enhancing the stability and security of their children and even the entire family, enabling them to better cope with environmental changes [31,32]. Therefore, we speculate that parental autonomy support may also affect the family’s “Double Reduction” adaptation through parent–child cohesion. However, there is currently no research exploring whether parental autonomy support can affect the entire family’s adaptation to the “Double Reduction” reform through parent–child cohesion.

### 1.4. Chain Mediation of Parent–Child Communication and Parent–Child Cohesion

In addition, in the context of “Double Reduction”, the impact of parental autonomy support on family adaptation may mainly be achieved through parent–child interaction. A good parent–child interaction model can form intimate family relationships and to some extent solve the problem of adaptation to stressful environments from the source [33]. In the family function theory model, it is proposed that parent–child communication and parent–child cohesion are the interaction mechanisms of parent–child behavior and emotion, respectively. The two play an important and special role in good family function [10]. Moreover, the interaction in parent–child behavior can further promote the emotional interaction between parents and children [34]. Empirical studies have shown that in the family system, positive parent–child communication and high-quality communication effects can help establish emotional connections between parents and children and enhance the cohesion and intimacy of the entire family [35]. Therefore, in the context of “Double Reduction”, parent–child communication and parent–child cohesion may play a chain mediating role between parental autonomy support and family adaptation. Parents’ autonomy support for their children helps promote positive communication between parents and children, thereby strengthening emotional connections between them. Families with high parent–child intimacy and cohesion are more able to adapt to the challenges and pressures under the context of the “Double Reduction” policy. On this basis, it is necessary to simultaneously pay attention to the quality of parental education methods and parent–child interaction to comprehensively and systematically reveal the impact mechanism of family adaptation under the “Double Reduction” policy.

### 1.5. The Present Study

**Figure 1:**
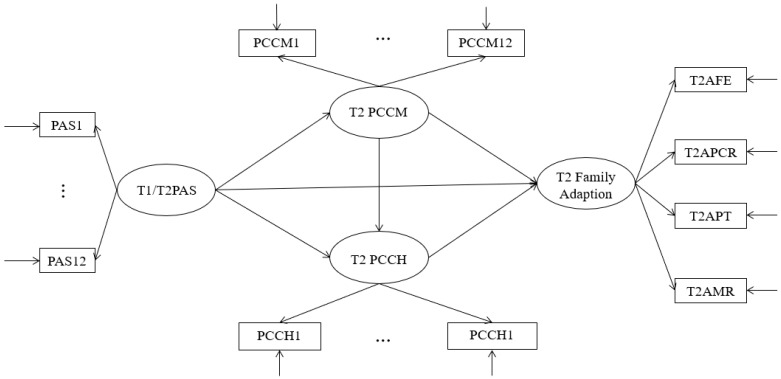
Hypothetical model. Note. PAS = parental autonomy support, PCCH = parent–child cohesion, PCCM = parent–child communication, AFE = adaptation to family education, APCR = adaptation of parent–child relationship, APT = adaptation of parents themselves, AMR = adaptation of marital relationship.

The purpose of this study was to investigate the impact and mechanism of parental autonomy support on family adaptation under the background of “Double Reduction” in China by using a chain mediation model and to take parent–child communication and parent–child cohesion as mediating variables from the perspective of parent–child behavior and emotion. The proposed model is presented in Figure 1.

Based on this, we put forward the following hypotheses: under the background of “Double Reduction”, (1) parental autonomy support positively predicts family adaptation, (2) parent–child communication plays a mediating role in parental autonomy support and family adaptation, (3) parent–child affinity plays a mediating role in parental autonomy support and family adaptation, and (4) parent–child communication and parent–child cohesion have a chain mediating role in parental autonomy support and family adaptation.

## 2. Method

### 2.1. Participants and Procedures

The study included 4239 parents (1493 fathers and 3427 mothers) in Zhejiang Province, China, with an average age of 43.20 years. The study, which was conducted in December 2022, required participants to complete a questionnaire based on their family’s adaptation one year after the implementation of the “Double Reduction” policy. To better understand the role of parental autonomy support on family adaptation, the study also used a retrospective method to require participants to recall the characteristics before the “Double Reduction” policy and complete the questionnaire.

The study has been approved by the School Ethics Committee. All materials and procedures of this study were approved by the Institute Review Board (IRB) of Zhejiang Normal University; the ethical code is D2020009. Before the official test, the consent of the teacher and the parents has been obtained. During the test, all participants completed the questionnaire under the guidance of a professionally trained examiner and a class teacher. The questionnaire was filled out via mobile phone, and participants were informed that the results will be treated confidentially.

### 2.2. Measures

#### 2.2.1. Family Adaptation 

The self-compiled Family Adaptation Scale under the implementation of the “Double Reduction” policy was adopted, with a total of 45 items. The items were categorized into four dimensions: adaptation of family education (e.g., “I am worried that I may not be able to provide necessary support for my child’s future”), adaptation of the parent–child relationship (e.g., “My communication with children is easy and harmonious”), adaptation of the parents themselves (e.g., “I feel a lot of pressure”), and adaptation of the marital relationship (e.g., “I will take the initiative to discuss the matter of my child with my spouse”). The items were scored on a 5-point scale, with 1 representing “Totally false” and 5 representing “Totally true”. The higher the score on the scale, the better the family adapts to the “Double Reduction”. In this study, the confirmatory factor analysis of the questionnaire showed that the construct validity of the questionnaire was acceptable (CFI = 0.90, TLI = 0.89, RMSEA = 0.04, SRMR = 0.11), and the Cronbach’s α coefficient of the scale was 0.87. 

#### 2.2.2. Parental Autonomy Support 

Wang et al. [36] developed the Parental Autonomy Support Scale with a total of 12 items (e.g., “When I have a problem, parents listen to my opinions and perspectives”). The items are scored on a 5-point scale, with 1 representing “Totally false” and 5 representing “Totally true”. A higher score on the scale indicates a higher level of parental autonomy support. The scale has good reliability and validity in Chinese applications (Tang et al., 2013). In this study, the Cronbach’s α coefficients of the T1 and T2 scales were 0.94 and 0.94.

#### 2.2.3. Parent–Child Communication

The Parent–Child Communication Quality Scale of the Parent–Child Communication Scale developed by Chi [20] was used with a total of 12 items (e.g., “My communication with my child makes each other closer”). The items are assessed using a 4-point scale, with 1 representing “Totally false” and 4 representing “Totally true”. The higher the score on the scale, the higher the quality of parent–child communication. The scale has good reliability and validity in Chinese applications [20]. In this study, the Cronbach’s α coefficient of the scale was 0.84.

#### 2.2.4. Parent–Child Cohesion

The parent–child cohesion questionnaire developed by Olson et al. [15] and revised by Wang and Zhang [37] was used to have a total of 10 items, and the items were scored on a 5-point scale, with 1 representing “never” and 5 representing “always”. Self-reported by the students’ parents, the degree of parent–child cohesion between parents and students was measured, and the higher the score of the questionnaire, the higher the degree of parent–child cohesion. In this study, the Cronbach’s α coefficient of the scale was 0.81.

### 2.3. Data Analysis 

Firstly, SPSS was used to identify descriptive statistics for each variable and calculate the correlation coefficient to investigate the correlation between the variables. Then, Mplus 8.0 was used to construct a structural equation model; the bootstrap method was used to test the mediating role of parent–child communication and parent–child cohesion, and age, gender, and family economic level were used as control variables. Standardized indirect effect parameters were calculated using 5000-replicate bootstrapping analyses. If the 95% confidence interval does not contain a zero, the indirect effect is noticeable.

For missing values present in the study data, we performed Little’s MCAR test before interpolating the missing values using the expectation maximization (EM) method [38]. Model fit was tested using the comparative fit index (CFI), Tucker–Lewis index (TLI), root mean square error of approximation (RMSEA), and standardized root mean square residual (SRMR). According to the suggested criteria [39], CFI and TLI ≥ 0.90, RMSEA ≤ 0.08, and SRMR ≤ 0.08 indicated a good model fit.

## 3. Result

### 3.1. Common Method Bias

To test for common method bias in this study, we used the Harman univariate method to test the data. The results showed that 10 factors (>2) had eigenvalues greater than 1 in the T1 period data, and the total variance explained by the first common factor was 27.25% (<40%). There are 13 factors (>2) with eigenvalues greater than 1 in the T2 period data, and the variance explained by the first major factor is 22.15% (<40%), indicating that there is no serious problem of common method bias in this study.

### 3.2. Description of Statistics and Comparison with the Current Situation

To examine the changes in parental autonomy support and family adaptation of middle school students before and after the “Double Reduction”, we first summed up parental autonomy support and the dimensions of family adaptation and then used a paired sample t test (see Table 1) to analyze whether there are differences in parental autonomy support and the four levels of family adaptation between middle school students before and after the “Double Reduction”. The results showed that the level of parental autonomy support after the “Double Reduction” was significantly higher than that before the “Double Reduction” (*p* < 0.001), and the family adaptation situation was also higher than before the “Double Reduction” (*p* < 0.001). In addition, in various dimensions of family adaptation, adaptation to family education, parent–child relationship, parental self adaptation, and marital relationship adaptation were significantly higher than those before the “Double Reduction” (*ps* < 0.001).

### 3.3. Related Analysis

First, we summed up the items of each variable before conducting correlation analysis. The sum, standard deviation, and correlation matrix of each variable are shown in Table 1. The correlation analysis results indicate that there is a significant positive correlation between parental autonomy support before and after “Double Reduction” (T1, T2) and various dimensions of family adaptation before and after “Double Reduction” (T1, T2). After the “Double Reduction” (T2), there were significant pairwise correlations between parental autonomy support and various dimensions of family adaptation, parent–child communication, and parent–child cohesion. Some demographic variables are significantly correlated with parental autonomy support, family adaptation, parent–child communication, and parent–child cohesion at two time points (subsequent model construction and testing will include demographic variables such as student age, gender, and the socioeconomic status of the family as covariates in the model for statistical control). The results are shown in Table 2.

### 3.4. Intermediary Analysis

**Figure 2:**
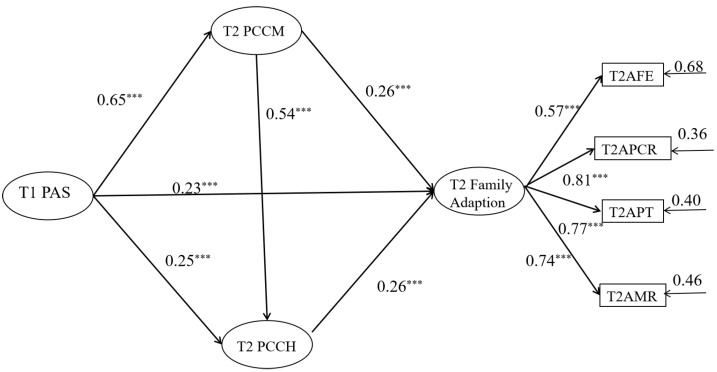
Parent–child communication and parent–child cohesion mediates the relationship between T1 parental autonomy support and T2 family adaptation. All coefficients in the notes are standardized estimates. Notes: The insignificant control variables were not shown here to simplify the representation of the model (similarly hereinafter). *** p < 0.001.

**Figure 3:**
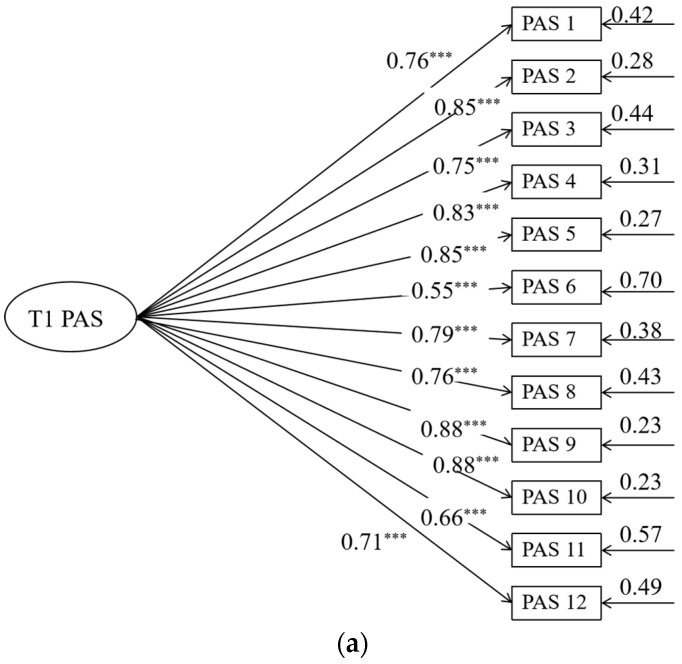

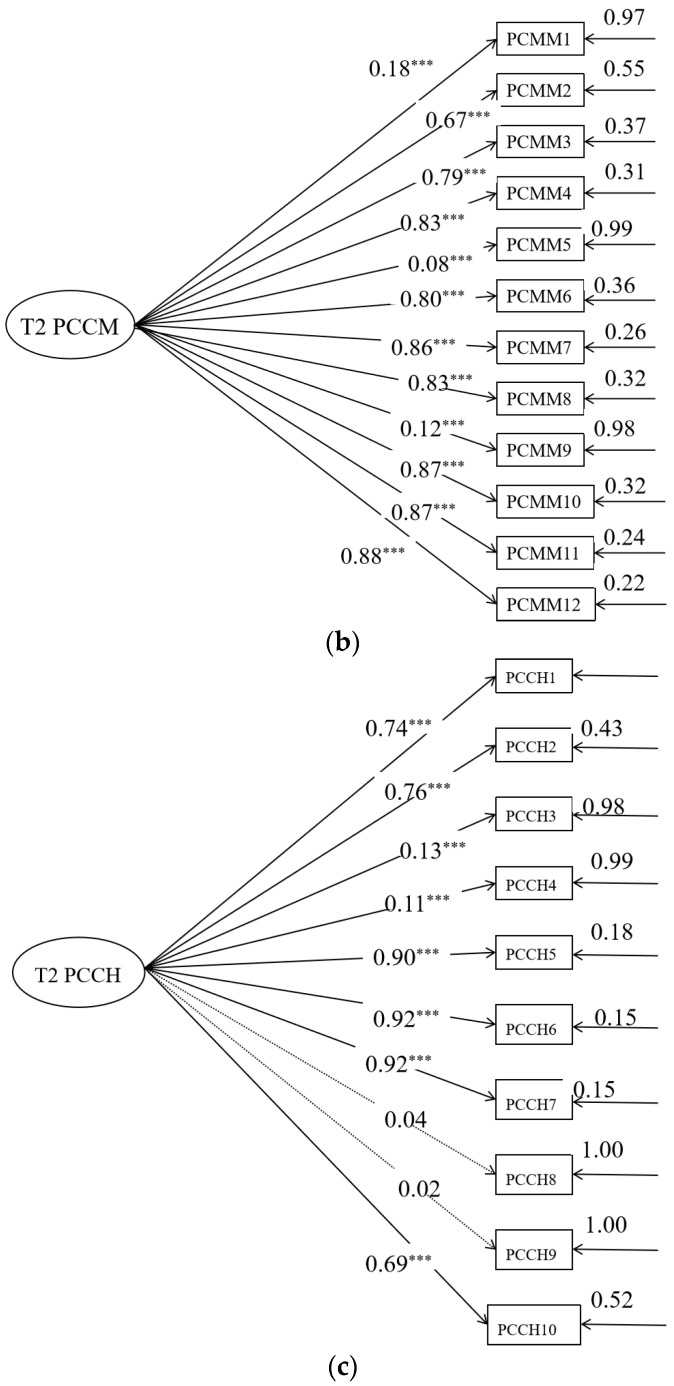
(**a**) The measurement model of parental autonomy support tested before “Double Reduction”. The circle represents the latent variable. A square represents an indicator. For simplicity, nonsignificant residuals were not described. The value on the longer single arrow represents the load value. The value of the smaller single arrow represents the residual variance. All displayed coefficients are standardized and reach statistical significance at the 0.05 level. *** *p* < 0.001. (**b**) The measurement model of parent–child communication tested after “Double Reduction”. The circle represents the latent variable. A square represents an indicator. For simplicity, nonsignificant residuals were not described. The value on the longer single arrow represents the load value. The value of the smaller single arrow represents the residual variance. All displayed coefficients are standardized and reach statistical significance at the 0.05 level. *** *p* < 0.001. (**c**) The measurement model of parent–child cohesion tested after “Double Reduction”. The circle represents the latent variable. A square represents an indicator. For simplicity, nonsignificant residuals were not described. The value on the longer single arrow represents the load value. The value of the smaller single arrow represents the residual variance. All displayed coefficients are standardized and reach statistical significance at the 0.05 level. *** *p* < 0.001.

To examine the relationship between parental autonomy support and family adaptation in the context of “Double Reduction”, as well as the role of parent–child communication and parent–child cohesion, we constructed a chain mediation model based on relevant analysis. In this model, parental age, gender, and the socioeconomic status of the family are used as control variables. The models are shown in Figure 2 and Figure 3.

The model in Figure 2 shows good fit: *χ*^2^ (756) = 16.64, *p* < 0.001, RMSEA (90% CI) = 0.06 (0.03~0.06), CFI = 0.91, TLI = 0.90, and SRMR = 0.06. As shown in Figure 2, after controlling for the age, gender, and socioeconomic status of the family of parents of junior high school students, parental autonomy support before “Double Reduction” significantly positively predicted parent–child communication, parent–child cohesion, and family adaptation after “Double Reduction” (*β =* 0.65, *p <* 0.001; *β =* 0.25, *p <* 0.001; *β =* 0.23, *p <* 0.001). Path analysis shows that parent–child communication significantly positively predicts parent–child cohesion and family adaptation after “Double Reduction” (*β =* 0.54, *p <* 0.001; *β =* 0.26, *p <* 0.001), as well as offering a significant positive prediction of parent-child cohesion for family adaptation after “Double Reduction” (*β =* 0.26, *p <* 0.001). The results indicate that parent–child communication may play a partial mediating role between parental autonomy support before “Double Reduction” and family adaptation after “Double Reduction”, and parent–child cohesion may play a partial mediating role between parental autonomy support before “Double Reduction” and family adaptation after “Double Reduction”. In addition, parent–child communication and parent–child cohesion may play a chain mediating role between parental autonomy support before “Double Reduction” and family adaptation after “Double Reduction”.

To further examine and analyze the mediating effect, the bootstrap method was used for indirect effect estimation, with 5000 replicates performed and a 95% confidence interval calculated. As shown in Table 3, both the direct and total indirect effects of parental autonomy support before “Double Reduction” and family adaptation after “Double Reduction” were significant (direct effects = 0.23, *p <* 0.01; indirect effects = 0.33, *p <* 0.01). Finally, an analysis was conducted on the mediating effects of each pathway, and the results showed that the 95% confidence interval of these pathways did not include zero, indicating that the aforementioned mediating pathways were significant. This means that parent–child communication and parent–child cohesion partially mediate the relationship between parental autonomy support and family adaptation. Meanwhile, parent–child communication and parent–child cohesion also play a chain mediating role between parental autonomy support before “Double Reduction” and family adaptation after “Double Reduction”.

**Figure 4:**
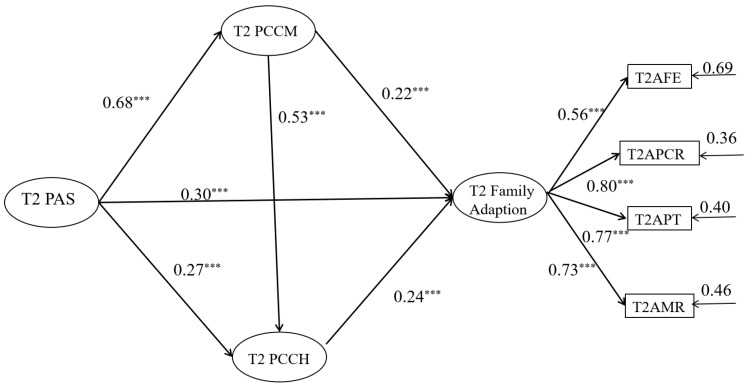
Parent–child communication and parent–child cohesion mediates the relationship between T1 parental autonomy support and T2 family adaptation. All coefficients in the notes are standardized estimates. *** *p* < 0.001.

**Figure 5:**
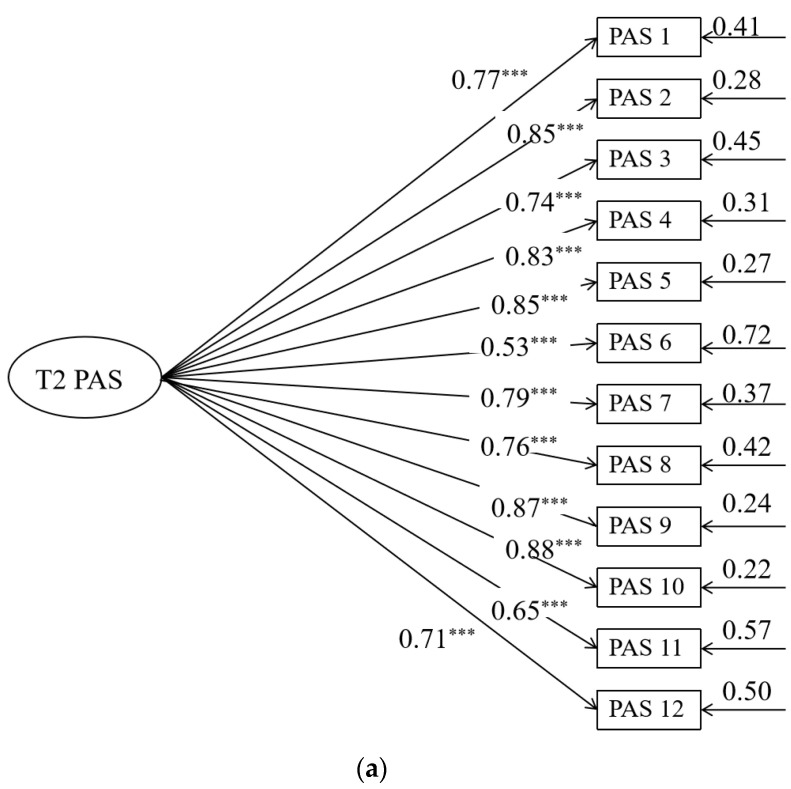

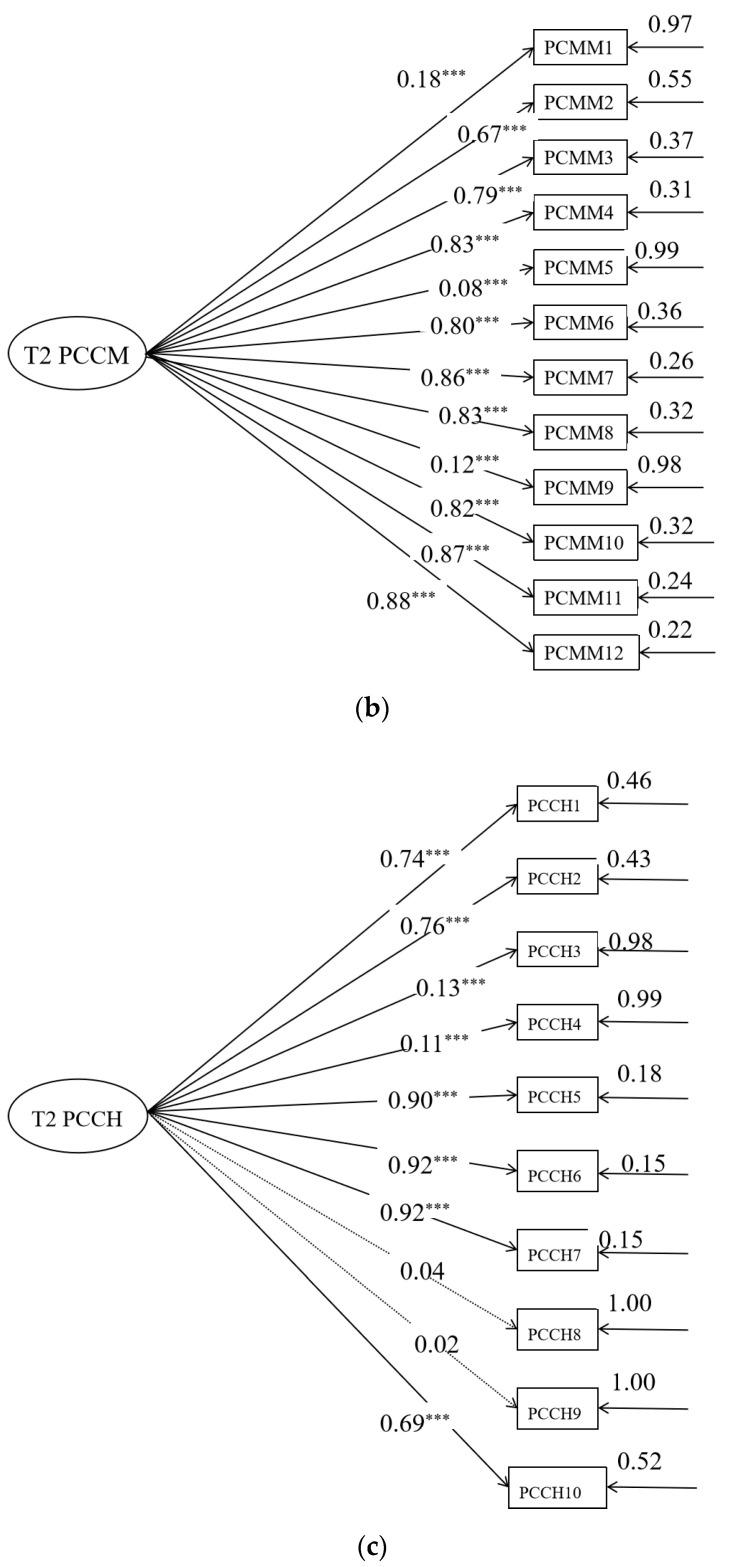
(**a**) The measurement model of parental autonomy support tested after “Double Reduction”. The circle represents the latent variable. A square represents an indicator. For simplicity, nonsignificant residuals were not described. The value on the longer single arrow represents the load value. The value of the smaller single arrow represents the residual variance. All displayed coefficients are standardized and reach statistical significance at the 0.05 level. *** *p* < 0.001. (**b**) The measurement model of parent–child communication, and parent–child cohesion tested after “Double Reduction”. The circle represents the latent variable. A square represents an indicator. For simplicity, nonsignificant residuals were not described. The value on the longer single arrow represents the load value. The value of the smaller single arrow represents the residual variance. All displayed coefficients are standardized and reach statistical significance at the 0.05 level. *** *p* < 0.001. (**c**) The measurement model of parent–child cohesion tested after “Double Reduction”. The circle represents the latent variable. A square represents an indicator. For simplicity, nonsignificant residuals were not described. The value on the longer single arrow represents the load value. The value of the smaller single arrow represents the residual variance. All displayed coefficients are standardized and reach statistical significance at the 0.05 level. *** *p* < 0.001.

The model in Figure 4 shows good fit: *χ*^2^ (756) = 16.54, *p <* 0.001, RMSEA (90% CI) = 0.06 (0.03~0.06), CFI = 0.91 TLI = 0.90, and SRMR = 0.06. The measurement model is shown in Figure 5. As shown in Figure 3, after controlling for the age, gender, and socioeconomic status of the family of parents of junior high school students, parental autonomy support after “Double Reduction” significantly positively predicted parent–child communication, parent–child cohesion, and family adaptation after “Double Reduction” (*β =* 0.68, *p <* 0.001; *β =* 0.27, *p <* 0.001; *β =* 0.30, *p <* 0.001). Path analysis shows that parent–child communication significantly positively predicts parent–child cohesion and family adaptation after “Double Reduction” (*β =* 0.53, *p <* 0.001; *β =* 0.22, *p <* 0.01), as well as offering a significant positive prediction of parent–child cohesion for family adaptation after “Double Reduction” (*β =* 0.24, *p <* 0.001). The results indicate that parent–child communication may play a partial mediating role between parental autonomy support and family adaptation after “Double Reduction”. Meanwhile, parent–child cohesion may play a partial mediating role between parental autonomy support and family adaptation after “Double Reduction”. In addition, parent–child communication and parent–child cohesion may play a chain mediating role between parental autonomy support and family adaptation after “Double Reduction”.

To further examine and analyze the mediating effect, the bootstrap method was used for indirect effect estimation, with 5000 replicates performed and a 95% confidence interval calculated. As shown in Table 4, the 95% confidence interval of these paths does not include zero, and like the model in Figure 2, the mediating paths are all significant. That is to say, both before and after the “Double Reduction”, parental autonomy support will significantly affect the family adaptation. Moreover, parent–child communication and parent–child cohesion can both play a partial mediating role and a chain mediating role between parental autonomy support and family adaptation.

## 4. Discussion

In the context of China’s “Double Reduction” policy, this study for the first time explores the direct effect and mechanism of parental autonomy support on family adaptation from the behavioral and emotional perspectives of parent–child interaction. The research results indicate that parental autonomy support can predict family adaptation. In addition, parental autonomy support can also influence the whole family’s adaptation through the quality of parent–child interaction. The present study provides evidence of scientific research at the family level for China’s “Double Reduction” policy over the past two years and provides the theoretical basis for family adaptation intervention.

### 4.1. Impact of Parental Autonomy Support on Family Adaptation 

This study found that parental autonomy support has a direct impact on family adaptation in the context of China’s “Double Reduction” policy. Specifically, the higher the level of autonomy support of parents before and after the “Double Reduction”, the better the family adaptation. The findings enriched relevant studies on parental autonomy support at the family level. Chinese families have always been in the hands of parents because of educational expectations such as the “Hope for sons to become dragons and daughters to become phoenixes.” The implementation of the “Double Reduction” has brought education back to being “student-centered” and has promoted the healthy development of students [40]. Parental autonomy support encourages children to make independent choices, cultivates children’s sense of self-awareness and psychological freedom, and creates a good and warm family atmosphere [41,42], and families can be in a free and relaxed atmosphere and better adapt to the changes brought about by the “Double Reduction”. As an external pressure on the family, “Double Reduction” is both a challenge and an opportunity. Under the impetus of the “Double Reduction” background, parents can reflect on their parenting concepts, create an autonomy-supporting family education environment, which can promote the family’s adaptation to the environment.

Not only that but the allocation of family education resources and prevailing economic conditions may be pivotal factors influencing parental behavior and family adaptation. Research suggests that social policy reforms not only reshape educational content, methodologies, and circumstances but also engender shifts in individuals’ educational ideologies [43]. The implementation of the “Double Reduction” policy facilitates a more equitable distribution of educational resources among students, thereby affording broader access to high-quality educational amenities within schools. Furthermore, this policy may exert influence on the financial inflows and outflows of households, consequently impacting the extent and manner in which parents invest in their children’s education. Preceding the introduction of the “Double Reduction” policy, intensified learning endeavors and extracurricular pursuits engendered cutthroat competition and excessive academic burdens among students vying for admission to institutions renowned for superior educational standards [44], thereby imposing substantial academic and financial strains on students and their families. The stringent prohibition on tutoring enforced by the “Double Reduction” policy has alleviated the financial strain on parents. However, the persisting pressure and fierce competition among students might continue to shape parental educational behaviors.

### 4.2. The Mediating Role of Parent–Child Communication

Our research findings indicate that parental autonomy support can indirectly influence family adaptation in the context of “Double Reduction” through parent–child communication. Parents provide their children with more autonomy and support, and the better the quality of parent–child communication, the better the entire family can adapt to the changes in education reform. This result is similar to previous studies on family adaptation and supports the family system theory [18,24]. The family system theory points out that the mode and quality of parent–child interaction are the core of the internal operation of the family. The parenting style of parents mainly affects the adaptation of the entire family through parent–child interaction. Supportive parenting behavior can improve the quality of parent–child communication and thus enhance family adaptation [19]. Before the implementation of the “Double Reduction” policy, parental autonomy support meant being more able to pay attention to children’s needs and emotional expression, giving children more autonomy space, which helped create a positive communication atmosphere [45]. This positive communication atmosphere may lay a good foundation for the quality of parent–child communication after the implementation of the “Double Reduction” policy, while also promoting understanding, support, and cooperation among family members [46], thereby helping families better adapt to changes in education policies. After the implementation of the “Double Reduction” policy, parental autonomy support still plays an important role. The “Double Reduction” policy’s guiding principles are to create a positive learning environment and support students’ overall development and well-being. As an indispensable educational role, parental autonomy support can meet the autonomous needs of students, help them cope with increased academic stress, and thus build a positive and warm communication environment [47,48]. This may further lead to a balanced, harmonious, and coherent state of family stress, enhancing the adaptability of families to the “Double Reduction” policy.

### 4.3. The Mediating Role of Parent–Child Cohesion

In addition, our research findings also indicate that parental autonomy support can indirectly affect family adaptation in the context of “Double Reduction” through parent–child cohesion. This result is similar to previous research findings [31,32]. That is to say, regardless of the situation, the autonomous support provided by parents to their children can enhance parent–child cohesion, improve parent–child relationships, make them more intimate, and thus enhance the family’s adaptability to sudden changes in the educational environment. This may be because supporting autonomous parenting provides a safe and warm environment for children, which is conducive to forming good parent–child relationships and promoting the harmonious development of the entire family [49]. This kind of good intimate relationship provides family members with a sense of security and helps them adapt to the stress and changes brought about by the “Double Reduction” policy [50]. The mediating role of parent–child cohesion between parental support and family adaptation will not change with the implementation of the “Double Reduction” policy. This is likely because the foundation of parent–child cohesion does not change, as it is based on a high density of relationships and emotional support among family members [51]. Regardless of the existence of the “Double Reduction” policy, parental autonomy support for children is an important factor affecting parent–child relationships and intimacy [52], and the mediating role of parent–child cohesion is also based on this level of family intimacy. Therefore, under the implementation of the “Double Reduction” policy, the mediating role of parent–child cohesion between parental autonomy support and family adaptation will not be affected and will not change.

### 4.4. Chain Mediation of Parent–Child Communication and Parent–Child Cohesion

It is worth noting that our study also found that parent–child communication and parent–child cohesion play a chain mediating role between parental autonomy support and family adaptation in the context of “Double Reduction”. This result supports the family system theory [10], which suggests that parent–child communication plays an important role in promoting parent–child cohesion. A good communication environment can promote cohesion between parents and children, deepen parent–child relationships, and thus enhance the family’s adaptability to stressful environments. Previous studies have found that adolescents who grow up in a family environment with high cohesion benefit from an intimate family atmosphere and are more adaptable in cognitive and social development [53]. Similarly, we speculate that the entire family will also benefit from a good communication environment and an intimate family atmosphere, making them more adaptable in the face of educational reform. This may be because when family members have a higher quality of parent–child interaction, they perceive life events from a relatively positive perspective. Even though the “Double Reduction” policy may be a sudden event for some families [1], it can still be adopted in a timely manner for families with good parent–child relationships to maintain a healthy family operation [54]. In addition, the transformation of parents’ educational concepts is the key to achieving the adaptation of the “Double Reduction” policy [55]. Parents who adopt long-term independent and supportive education methods lead to an improvement in the quality of parent–child communication, resulting in a strengthening of parent–child cohesion and an increase in opportunities for interaction and emotional exchange between parents and children, resulting in a closer parent–child relationship. Parents at the center of change can better adapt to the new changes and requirements of the “Double Reduction” policy. Therefore, parents should strive to create a warm and loving family atmosphere, enhance parent–child interaction, and improve the quality of parent–child companionship to enhance the adaptability of families to the “Double Reduction” policy.

### 4.5. Limitations and Implications

The current study has certain limitations, just like any other research. First, the data in this study were derived from self-report questionnaires. Measurement data may be biased due to societal expectations and individual subjective factors. A wider variety of research methods can be used in the future. Secondly, due to the unpredictability of the policy, this study used a retrospective method to collect the data before the “Double Reduction”. The measurement data may contain recall bias or confuse other unknown factors. More controls can be put in place in the future to ensure greater validity and accuracy of the data. Third, this study has cultural adaptability, and the “Double Reduction” policy is targeted at China’s national conditions. Therefore, the results of this study may have certain limitations in generalizing to other countries where education policies have changed.

Nevertheless, our research still holds significant importance. Firstly, this study enriches the research on the implementation effect of China’s “Double Reduction” policy, with a focus on the family dimension. The “Double Reduction” policy is a significant educational reform in China that has an impact on Chinese educational development as well as hundreds of millions of families. Secondly, this study further expands the research on family-based parental autonomy support and enriches its practical significance. No matter when parents start providing autonomous and supportive education to their children, it is never too late and can promote the adaptation of the entire family. Finally, this study explores in detail the specific mechanisms by which family adaptation affects “Double Reduction” from the perspectives of parent–child behavior and emotions. This provides us with a new perspective on promoting parent–child relationships, family adaptation, and joint education between family, school, and society. For example, when calling on families to respond to the “Double Reduction” policy, the government and schools can emphasize the importance of the overall adaptation of families to changes, advocate for parental autonomy and support, and pay attention to communication quality and emotional connection to improve parent–child relationships.

## 5. Conclusions

In the context of “Double Reduction”, parental autonomy support can directly predict family adaptation. Secondly, parent–child communication and parent–child fit can mediate the relationship between parental autonomy support and family adaptation, respectively. Finally, parent–child communication and cohesion play a chain mediating role between parental autonomy support and family adaptation.

## Figures and Tables

**Table 1:** Test for differences in various dimensions of family adaptation before and after “Double Reduction”.

Variables	Before “Double Reduction” (T1)	After “Double Reduction” (T2)	*t*
Parental Autonomy Support	3.82 ± 0.55	3.85 ± 0.55	−10.03 ***
AFE	3.02 ± 0.41	3.02 ± 0.42	−3.49 ***
APCR	3.66 ± 0.44	3.69 ± 0.44	−11.19 ***
APT	3.40 ± 0.57	3.42 ± 0.58	−8.37 ***
AMR	3.61 ± 0.55	3.63 ± 0.56	−9.52 ***
Family Adaptation	3.36 ± 0.38	3.38 *±* 0.39	−9.95 ***

Note. AFE = adaptation to family education, APCR = adaptation of parent–child relationship, APT = adaptation of parents themselves, AMR = adaptation of marital relationship *** *p* < 0.001.

**Table 2:** Means (*M*s), standard deviations (*SD*s), and bivariate correlations of key study variables.

	*M ± SD*	1	2	3	4	5	6	7	8	9	10	11	12	13	14	15
1. T1 PAS	3.82 ± 0.55	1														
2. T2 PAS	3.85 ± 0.55	0.95 ***	1													
3. T1 AFE	3.02 ± 0.41	0.26 ***	0.26 ***	1												
4. T1 APCR	3.66 ± 0.44	0.53 ***	0.52 ***	0.40 ***	1											
5. T1 APT	3.40 ± 0.57	0.39 ***	0.39 ***	0.51 ***	0.60 ***	1										
6. T1 AMR	3.61 ± 0.55	0.42 ***	0.42 ***	0.38 ***	0.59 ***	0.57 ***	1									
7. T2 AFE	3.02 ± 0.42	0.25 ***	0.27 ***	0.95 ***	0.39 ***	0.49 ***	0.38 ***	1								
8. T2 APCR	3.69 ± 0.44	0.50 ***	0.54 ***	0.38 ***	0.92 ***	0.56 ***	0.58 ***	0.42 ***	1							
9. T2 APT	3.42 ± 0.58	0.37 ***	0.43 ***	0.49 ***	0.56 ***	0.94 ***	0.56 ***	0.37 ***	0.61 ***	1						
10. T2 AMR	3.63 ± 0.56	0.40 ***	0.44 ***	0.37 ***	0.57 ***	0.55 ***	0.97 ***	0.42 ***	0.60 ***	0.58 ***	1					
11. T2PCCM	2.92 ± 0.41	0.58 ***	0.61 ***	0.40 ***	0.56 ***	0.47 ***	0.49 ***	0.52 ***	0.59 ***	0.49 ***	0.51 ***	1				
12. T2PCCH	3.60 ± 0.57	0.44 ***	0.46 ***	0.36 ***	0.52 ***	0.43 ***	0.48 ***	0.40 ***	0.54 ***	0.45 ***	0.49 ***	0.67 ***	1			
13. Age	43.202 ± 22.39	−0.01	−0.01	0.02	−0.00	0.00	−0.00	0.02	0.00	0.01	0.00	−0.01	−0.01	1		
14. Gender	/	0.04 **	0.03 *	0.10 ***	0.05 ***	0.05 ***	0.01	0.08 ***	0.03 *	0.07 ***	0.01	0.05 ***	0.12 ***	−0.05 **	1	
15. SES	/	0.17 ***	0.18 ***	0.22 ***	0.18 ***	0.28 ***	0.19 ***	0.21 ***	0.17 ***	0.27 ***	0.19 ***	0.17 ***	0.12 ***	−0.03	0.06 ***	1

Note. PAS = parental autonomy support, AFE = adaptation to family education, APCR = adaptation of parent–child relationship, APT = adaptation of parents themselves, AMR = adaptation of marital relationship, PCCM = parent–child communication, PCCH = parent–child cohesion, SES = Socioeconomic status of the family. * p < 0.05, ** *p* < 0.01, *** *p* < 0.001. Same as below.

**Table 3:** Standardized path coefficients of T1 parental autonomy support, parent–child communication, T2 parent–child cohesion, and T1 family adaptation.

Effects	Model Pathways	Effect Value	95% CI	
			Lower	Upper
Direct effect	T1 parental autonomy support → T2 family adaptation	0.227 ***	0.184	0.271
Mediating effects	T1 parental autonomy support → T2 parent–child communication → T2 family adaptation	0.168 ***	0.134	0.198
	T1 parental autonomy support→T2 parent–child cohesion → T2 family adaptation	0.066 ***	0.054	0.082
	T1 parental autonomy support → T2 parent–child communication → T2 parent–child cohesion → T2 family adaptation	0.093 ***	0.080	0.109
	Total mediating effect	0.327 ***	0.299	0.356
Total effect		0.554 ***	0.521	0.584

Note. All the coefficients are standardized estimates (similarly hereinafter). *** *p <* 0.001.

**Table 4:** Standardized path coefficients of T2 parental autonomy support, parent–child communication, T2 parent–child cohesion, and T2 family adaptation.

Effects	Model Pathways	Effect Value	95% CI	
			Lower	Upper
Direct effect	T2 parental autonomy support → T2 family adaptation	0.303 ***	0.259	0.341
Mediating effects	T2 parental autonomy support → T2 parent–child communication → T1 family adaptation	0.147 ***	0.115	0.177
	T2 parental autonomy support → T1 parent–child cohesion → T1 family adaptation	0.064 ***	0.051	0.079
	T2 parental autonomy support → T2 parent–child communication → T2 parent–child cohesion → T2 family adaptation	0.086 ***	0.072	0.102
	Total mediating effect	0.297 ***	0.270	0.328
Total effect		0.601 ***	0.573	0.628

*** *p* < 0.001.

## Data Availability

The data supporting this study’s findings are available from the corresponding author upon reasonable request.
